# Novel dipeptidyl peptidase‐IV and angiotensin‐I‐converting enzyme inhibitory peptides released from quinoa protein by in silico proteolysis

**DOI:** 10.1002/fsn3.1423

**Published:** 2020-01-27

**Authors:** Huimin Guo, Aurore Richel, Yuqiong Hao, Xin Fan, Nadia Everaert, Xiushi Yang, Guixing Ren

**Affiliations:** ^1^ Institute of Crop Science Chinese Academy of Agricultural Sciences Beijing China; ^2^ Gembloux Agro‐Bio Tech University of Liège Gembloux Belgium

**Keywords:** ACE inhibitors, bioactive peptides, DPP‐IV inhibitors, in silico approach, quinoa protein

## Abstract

Quinoa protein has been paid more and more attention because of its nutritional properties and beneficial effects. With the development of bioinformatics, bioactive peptide database and computer‐assisted simulation provide an efficient and time‐saving method for the theoretical estimation of potential bioactivities of protein. Therefore, the potential of quinoa protein sequences for releasing bioactive peptides was evaluated using the BIOPEP database, which revealed that quinoa protein, especially globulin, is a potential source of peptides with dipeptidyl peptidase‐IV (DPP‐IV) and angiotensin‐I‐converting enzyme (ACE) inhibitory activities. Three plant proteases, namely papain, ficin, and stem bromelain, were employed for the in silico proteolysis of quinoa protein. Furthermore, four tripeptides (MAF, NMF, HPF, and MCG) were screened as novel promising bioactive peptides by PeptideRanker. The bioactivities of selected peptides were confirmed using chemical synthesis and in vitro assay. The present work suggests that quinoa protein can serve as a good source of bioactive peptides, and in silico approach can provide theoretical assistance for investigation and production of functional peptides.

## Graphical abstract

Quinoa protein, especially globulin, is a potential source of bioactive peptides. In silico analysis makes a contribution to the exploration of novel bioactive peptide.

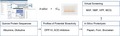

## 1 INTRODUCTION

In recent years, there is an increasing interest in food protein‐derived peptides for their diverse physiological activities such as antioxidant, angiotensin‐I‐converting enzyme (ACE) inhibitory, and dipeptidyl peptidase‐IV (DPP‐IV) inhibitory activities. Many studies have been focused on the use of food protein as raw materials for the production of bioactive peptides (Ghribi et al., 2015; Nongonierma, Lalmahomed, Paolella, & FitzGerald, 2017; Uraipong & Zhao, 2018; Venuste et al., 2013). Among researches that have been made, the digestion of protein is the limiting factor in the release of bioactive peptides, with the most common and effective method to be enzymatic hydrolysis. The conventional method in the discovery of novel bioactive peptides includes not only enzymolysis in vitro or in vivo, but also a complex series of subsequent steps, that is, separation, purification, and identification of peptides with given bioactivity. With the development of bioinformatics, in silico analysis has been greatly used to investigate the bioactive features of protein and peptides, which is more economical and time‐saving than conventional method. BIOPEP database, providing collection of sequences (proteins, bioactive peptides, allergenic proteins, and sensory peptides), can be used to predict biological activities about a protein sequence, and to estimate the release of bioactive peptides by proteolysis simulation using certain proteases (Minkiewicz, Dziuba, Iwaniak, Dziuba, & Darewicz, 2008). This in silico tools has been successfully applied in the investigation of bioactive peptides from different sources, including animal products, plant products, and seafood products, such as bovine meat proteins (Minkiewicz, Dziuba, & Michalska, 2011), porcine myofibrillar proteins (Kęska & Stadnik, 2016), yak milk casein (Lin et al., 2018), cereal storage proteins (Cavazos & de Mejia, 2013), oilseed proteins (Han, Maycock, Murray, & Boesch, 2019), giant grouper roe proteins (Panjaitan, Gomez, & Chang, 2018), and portuguese oyster proteins (Gomez, Peralta, Tejano, & Chang, 2019). In addition, online tool PeptideRanker has the function of predicting the potential bioactive index of peptides, and ToxinPred has been developed to predict the toxicity of peptides.

Quinoa (*Chenopodium quinoa* Willd.) is an ancient crop and has been recognized as a potent food candidate due to its exceptional nutritive value. There is now much interest in quinoa protein for its good balance of amino acids, gluten‐free property, and high digestibility (Filho et al., 2017; Graf et al., 2015). In addition to the nutritional value, quinoa protein has been documented to exert some beneficial effects as a source of bioactive peptides, like ACE inhibition (Aluko & Monu, 2003), antioxidant (Aluko & Monu, 2003; Nongonierma, Maux, Dubrulle, Barre, & FitzGerald, 2015), DPP‐IV inhibition (Nongonierma et al., 2015), antidiabetic (Vilcacundo, Martínez‐Villaluenga, & Hernández‐Ledesma, 2017), and colon cancer cell viability inhibitory effect (Vilcacundo, Miralles, Carrillo, & Hernández‐Ledesma, 2018).

However, the potential of quinoa protein to release biological peptides has not been studied systematically. The aim of the present work was to study the potential use of quinoa protein as the precursor of bioactive peptides based on in silico analysis, and to assess the potential of some enzymes to release bioactive peptides by enzymatic hydrolysis simulation. Furthermore, this in silico analysis was used for the exploration of novel bioactive peptides derived from quinoa protein.

## 2 MATERIALS AND METHODS

### 2.1 Protein sequences and enzymes

Five sequences of quinoa seed storage proteins were selected for the in silico analysis: 2S albumin‐like (XP_021758596), 11S seed storage globulin (AAS67036), 11S globulin seed storage protein 2‐like (XP_021770184), 13S globulin seed storage protein 1‐like (XP_021752233), and 13S globulin seed storage protein 2‐like (XP_021752668). Besides, soybean proteins, glycinin (P04347), β‐conglycinin α′ (P11827), and β‐conglycinin α (P0DO16), were taken as comparison sequences to assay the potential biological activity of different proteins. All sequence information was retrieved from NCBI (https://www.ncbi.nlm.nih.gov/) and listed in Table 1.

**Table 1:** Quinoa and soybean protein sequences used for in silico analysis in this study

Source	Protein	Accession (NCBI)	Length	Abbreviation
Quinoa	2S albumin‐like	XP_021758596	142	2S
	11S seed storage globulin	AAS67036	480	11S‐1
	11S globulin seed storage protein 2‐like	XP_021770184	474	11S‐2
	13S globulin seed storage protein 1‐like	XP_021752233	463	13S‐1
	13S globulin seed storage protein 2‐like	XP_021752668	542	13S‐2
Soybean	Glycinin	P04347	516	–
	β‐conglycinin α′	P11827	621	–
	β‐conglycinin α	P0DO16	605	–

In this study, three plant proteases were used for in silico proteolysis: papain (EC 3.4.22.2), ficin (EC 3.4.22.3), and stem bromelain (EC 3.4.22.32). Meanwhile, pepsin (pH > 2.0, EC 3.4.23.1), trypsin (EC 3.4.21.4), and chymotrypsin (EC 3.4.21.1) were employed to evaluate the stability of the peptides against gastrointestinal digestion.

### 2.2 Evaluation of quinoa proteins as a precursor of bioactive peptides via the BIOPEP database

Profiles for quinoa proteins as the precursor of bioactive peptides is available in the BIOPEP (http://www.uwm.edu.pl/biochemia/index.php/pl/biopep) using the “Profiles of potential biological activity” tool, shown as the type and location of bioactive fragment in a protein sequence. Meanwhile, the frequency of the occurrence of peptides with given activity (*A*) in a protein was taken as the evaluation parameter and calculated based on the equation:

(1) A=a/N

where a is the number of peptides with given activity in the protein sequence, and *N* is the number of amino acid residues in the protein. The total frequency of occurrence of all bioactive peptides (∑*A*) in the protein was also calculated.

### 2.3 In silico proteolysis and virtual screening

The proteolysis simulation provided by BIOPEP was adopted. Papain, ficin, and stem bromelain were independently applied to the protein sequences to release peptides. The frequency of release of peptides with given bioactivity by selected enzymes (*A*_E_) and the relative frequency of release of peptides with given activity by selected enzymes (*W*) were calculated according to the equations:

(2) $$A_{E}=d/N$$

where *d* is the number of peptides with given activity released from the protein sequence by selected enzyme, and N is the number of amino acid residues in the protein.

(3) $$W=A_{E}/A$$

Then, the peptides with three amino acids were submitted to PeptideRanker (http://distilldeep.ucd.ie/PeptideRanker/) for the calculation of theoretical bioactivity of peptides, and the results were presented as score values from 0 (poorest bioactivity) to 1 (best bioactivity). Peptides with relatively high PeptideRanker score and no previously described bioactivity based on the information recorded in BIOPEP database were evaluated for their stability against the gastrointestinal digestion using BIOPEP simulation, and their toxicity using ToxinPred (http://crdd.osdd.net/raghava/toxinpred/multi_submit.php). The solubility of the peptide was evaluated by the online Innovagen server, available at http://www.innovagen.com/proteomics-tools.

### 2.4 Peptide synthesis

Screened peptides were synthesized by the Sangon Biotech Company for the evaluation of in vitro DPP‐IV and ACE inhibitory activities. The purity of the peptide was 99% verified by HPLC.

### 2.5 Assay of DPP‐IV inhibitory activity

The DPP‐IV inhibition assay was determined using DPP‐IV inhibitor screening assay kit (KA1311, Abnova). Briefly, peptide samples (10 μl), dispersed in assay buffer (20 mM Tris‐HCl containing 100 mM NaCl and 1 mM EDTA, pH 8.0) at various concentrations, were mixed with assay buffer and DPP‐IV in a 96‐well plate. Then, substrate solution (Gly‐Pro‐Aminomethylcoumarin) was added to initiate the reactions. The mixture was incubated at 37°C for 30 min, and the fluorescence was measured using a plate reader (Synergy MX, Bio Tek) at an excitation wavelength of 350 nm and an emission wavelength of 450 nm. The concentration of the DPP‐IV inhibitor required to inhibit 50% of DPP‐IV activity under the above assay conditions was defined as the IC_50_, which was the mean value from three independent replicate assays.

### 2.6 Assay of ACE inhibitory activity

The ACE inhibition assay was carried out with the ACE inhibitory assay kit (ACE kit‐WST). Absorbance at 450 nm was measured using a plate reader (SpectraMax plus, Molecular devices), and the IC_50_ value reported for each sample was the mean value from three independent replicate assays.

### 2.7 Statistical analysis

All tests for peptides bioactivities were conducted with three replicates, and their data were expressed as the mean ± standard deviations. Statistical analysis was performed using SPSS version 16.0. Differences between the means were tested using one‐way ANOVA with Duncan's test. Mean values were considered significantly different at *p* < .01.

## 3 RESULTS AND DISCUSSIONS

### 3.1 The potential of quinoa seed storage protein as a precursor of bioactive peptides

Globulin and albumin were found to be dominant in quinoa seed protein (Brinegar, Sine, & Nwokocha, 1996; Prakash & Pal, 1998). To investigate the potential of quinoa protein as precursors of bioactive peptides, a total of five quinoa protein sequences with a range of 142–542 amino acids (Table 1 and Appendix S1) were selected and assessed by “Profiles of potential biological activity” of BIOPEP, and three soybean protein sequences as comparison. Soybean is an important crop in many countries for its high‐quality protein and kinds of biological activities. Glycinin and β‐conglycinin have been regarded as the good precursors of bioactive peptides (Han et al., 2019; Singha, Vij, & Hati, 2014).

**Figure 1:**
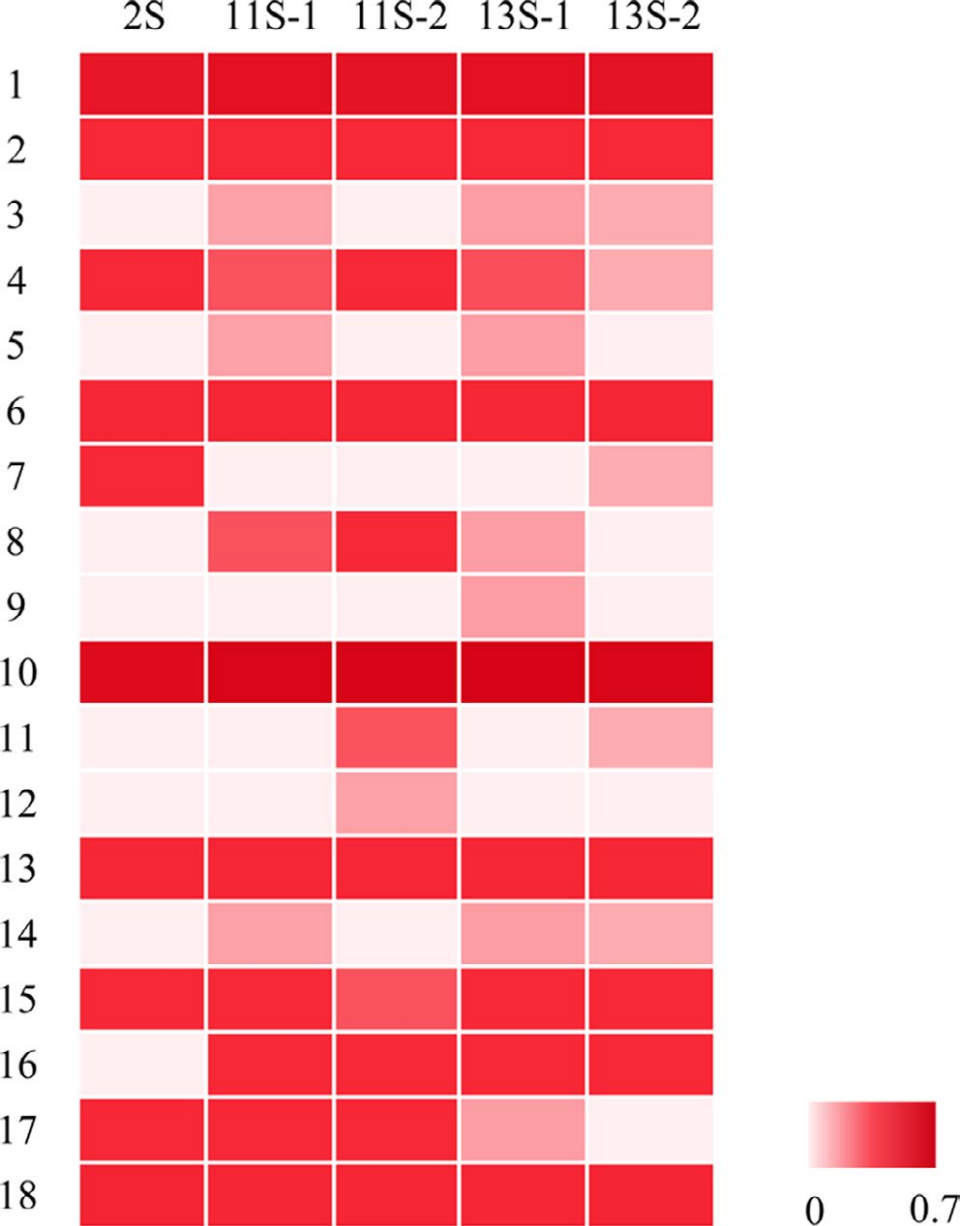
The frequency of the occurrence of peptides with given activity in quinoa protein performed by BIOPEP. (1. ACE inhibitor; 2. Peptide activating ubiquitin‐mediated proteolysis; 3. α‐glucosidase inhibitor; 4. Antiamnestic peptide; 5. Anticancer peptide; 6. Antioxidative peptide; 7. Calcium‐binding peptide; 8. Antithrombotic peptide; 9. Bacterial permease ligand; 10. DPP‐IV inhibitor; 11. Embryotoxic; 12. Hydroxy methylglutaryl coenzyme A reductase inhibitor; 13. Renin inhibitor; 14. Immunomodulating peptide; 15. CaMPDE inhibitor; 16. Neuropeptide; 17. Peptide regulating the stomach mucosal membrane activity; 18. Glucose uptake stimulating peptide.)

Based on the present limited information in BIOPEP database (as of 11 June 2019, 3,792 peptides functioned in 51 bioactivities have been collected in BIOPEP), fragments with 18 known biological activities were found in quinoa proteins (Figure 1). Among them, fragments with ACE inhibition, activating ubiquitin‐mediated proteolysis, antiamnestic, antioxidative, DPP‐IV inhibition, renin inhibition, inhibiting calmodulin‐dependent phosphodiesterase (CaMPDE), and stimulating glucose uptake activities existed in all analyzed quinoa protein sequences.

As for the total frequency of bioactive peptides occurrence, 11S‐2 (∑*A* = 1.2508) had the highest value of seven analyzed proteins, followed by 11S‐1 (∑*A* = 1.2480) and 13S‐1 (∑*A* = 1.2443). These three quinoa proteins showed higher total frequency of bioactive peptides occurrence than soybean proteins (Table 2). Quinoa albumin 2S had the weakest potential to act as precursor of bioactive peptides, with the least bioactivities and lowest occurrence frequency of bioactive peptides (∑*A* = 1.0139). Globulin is the principal precursor of bioactive peptides in quinoa seed.

**Table 2:** The frequency of occurrence of peptides with a given activity (*A*) in selected protein sequences

Source	Protein	Number of activities	∑*A*	*A*_1_	*A*_2_
				(DPP‐IV inhibitor)	(ACE inhibitor)
Quinoa	2S	9	1.0139	0.5211	0.3451
	11S‐1	14	1.2480	0.6354	0.4208
	11S‐2	13	1.2508	0.6540	0.3945
	13S‐1	15	1.2443	0.6609	0.4168
	13S‐2	13	1.2082	0.6181	0.3930
Soybean	Glycinin	13	1.1628	0.6182	0.3876
	β‐conglycinin α′	15	1.1949	0.5781	0.3833
	β‐conglycinin α	15	1.2100	0.5752	0.3934

DPP‐IV (*A*_(DPP‐IV inhibitor)_ = 0.5211–0.6609) and ACE inhibitor (*A*_(ACE inhibitor)_ = 0.3451–0.4208) were the major part of bioactive fragments in all selected protein sequences and were taken as the research focus in this paper. DPP‐IV is a ubiquitous protease associated with the degradation of incretin and regulation of blood glucose levels, and drug based on the inhibition of its activity is one of the most recent treatments for type 2 diabetes mellitus (Juillerat‐Jeanneret, 2014). Food protein‐derived DPP‐IV inhibitors have been intensively studied over the last few decades (Lacroix & Li‐Chan, 2012). ACE plays a significant role in blood pressure regulation by promoting the production of the active hypertensive hormone and inactivation of vasodilator peptide, making it one of the promising physiological targets for antihypertensive drugs (Miralles, Amigo, & Recio, 2018; Udenigwe & Mohan, 2014). Various dietary proteins have been employed for the generation of ACE inhibitory hydrolysates, including animal products, marine organisms, and plants (Lee & Hur, 2017).

The highest release frequency of DPP‐IV and ACE inhibitor was found in quinoa 13S‐1 and 11S‐1, respectively. Quinoa globulin 11S‐1, 11S‐2, and 13S‐1 exerted higher release frequency for DPP‐IV and ACE inhibitory peptides than analyzed soybean proteins. Frequency parameters of 13S‐2 were slightly lower than the highest value in soybean proteins. Our study demonstrated that globulin in quinoa seed presented a high potential as a precursor for the production of various biologically active peptides, especially DPP‐IV and ACE inhibitors.

### 3.2 In silico proteolysis of quinoa proteins

Bioactive peptides encrypted within the natural food protein are supposed to be released by enzymolysis to exert their biological function. A number of food processing enzymes were previously used for the generation of bioactive peptides from a variety of natural sources (Fu, Wu, Zhu, & Xiao, 2016; Lin, Zhang, Han, Meng, et al., 2018). In our study, three commercial plant proteases papain, ficin, and stem bromelain were applied, respectively, to the selected quinoa protein sequences by “Enzyme(s) action” of BIOPEP (Appendix S2). Hydrolysates with the degree of hydrolysis (DH) between 31.2925% and 52.1298% were obtained by in silico proteolysis (Table 3). Among the three enzymes, stem bromelain gave the highest DHs for five quinoa proteins, while the release of bioactive peptides is not proportional to the DH of hydrolysate.

**Table 3:** The parameters describing the predicted efficiency of release of bioactive fragments from quinoa protein by in silico enzymolysis

Protein	Enzymes	DH_t_ (%)	DPP‐IV inhibitor		ACE inhibitor	
			*A*_E_	*W*	*A*_E_	*W*
2S	Papain	35.3741	0.0541	0.1082	0.0270	0.0833
	Ficin	31.2925	0.0270	0.0540	0.0135	0.0416
	Stem bromelain	35.3741	0.0541	0.1082	0.0203	0.0626
11S‐1	Papain	43.6105	0.0850	0.1386	0.0506	0.1244
	Ficin	44.0162	0.0668	0.1089	0.0364	0.0895
	Stem bromelain	52.1298	0.0972	0.1585	0.0425	0.1044
11S‐2	Papain	39.0593	0.0735	0.1177	0.0408	0.1087
	Ficin	44.1718	0.0735	0.1177	0.0510	0.1358
	Stem bromelain	50.3067	0.0633	0.1014	0.0408	0.1087
13S‐1	Papain	42.0168	0.1027	0.1622	0.0566	0.1429
	Ficin	40.7563	0.0566	0.0894	0.0398	0.1005
	Stem bromelain	51.6807	0.0692	0.1093	0.0482	0.1217
13S‐2	Papain	40.0359	0.0663	0.1121	0.0376	0.0999
	Ficin	46.6786	0.0591	0.0999	0.0358	0.0951
	Stem bromelain	52.0646	0.0573	0.0969	0.0323	0.0858

The evaluation parameters (*A*_E_ and *W*) of DPP‐IV and ACE inhibitory peptides generated in this study were shown in Table 3. The release frequency (*A*_E_) of DPP‐IV inhibitory peptides was higher than that of ACE inhibitory peptides generated from the same sequence by the same enzyme, and the similar results were seen in the relative release frequency (*W*) of peptides except 11S‐2 and 13S‐1 treated by ficin and stem bromelain.

Different enzymes have different potential to release bioactive peptides from proteins, which attribute to their specific cleavage sites (Gomez et al., 2019). For example, Fu et al. (2016) performed in silico proteolysis of bovine collagen by twenty‐seven different enzymes and found that papain was the most effective protease to release ACE inhibitory peptides theoretically. In our study, papain‐treated quinoa proteins (except 11S‐1) exerted relatively higher release frequency index of DPP‐IV inhibitors than the other two enzymes. Similarly, papain has relative strong potential as an enzyme releasing ACE inhibitory peptides from quinoa proteins (except 11S‐2). This might be because papain shared most of the cutting sites with two other enzymes, except for those from the N‐terminus (Appendix S3).

The sequences of identified DPP‐IV and ACE inhibitory peptides predicted to be released from quinoa proteins by in silico enzymolysis were presented in Table 4. These bioactive peptides are made up of relatively few amino acids; exactly, most of them are dipeptides, except for IVR, IVY, AQL, VTR, and NKL. Actually, there were still plenty of peptides with no previously described bioactivity released from in silico enzymolysis of quinoa proteins. As for the bioactivity of the unknown peptides, further study is required.

**Table 4:** BIOPEP analysis of bioactive peptides predicted to be released from quinoa protein based on in silico enzymolysis with papain, ficin, and stem bromelain

	DPP‐IV inhibitors	ACE inhibitors
Papain	**171** a **:**	**96:**
	VV (1)^b^, SP (1), KP (1), NP (2), QP (1), HL (2), AL (11), SL (4), VR (2), PL (3), WI (1), YT (1), AD (2), AE (4), AF (5), AG (19), AH (1), AT (1), AY (1), DP (1), EG (3), EH (1), ES (1), ET (1), HR (2), HT (1), IH (1), IL (3), IR (2), KF (2), KG (1), KR (1), KT (4), MR (4), NF (1), NG (5), NL (1), NR (1), PF (1), PG (1), QD (1), QF (4), QG (17), QH (3), QI (1), QL (17), QN (1), QT (6), QV (1), QW (1), SF (4), VF (4), VL (3), VT (3), YL (3), YR (1)	IR (2), IY (1), VF (4), PR (1), YL (3), AY (1), IVR (1), PL (3), AF (5), KR (1), IF (1), AG (19), HL (2), KG (1), HG (1), QG (17), SG (6), EG (3), NG (5), PG (1), VR (2), NF (1), SF (4), KF (2), AR (3), KP (1), IE (1), AH (1), IL (3)
Ficin	**132:**	**83:**
	EK (1), AL (2), VR (4), PL (5), WR (1), AG (9), EG (4), EH (1), ES (6), EY (1), IH (1), IL (4), IR (3), MG (1), MK (2), MR (3), NF (1), NG (8), NH (1), NL (4), NR (1), NY (1), PF (3), PG (2), PH (1), PK (2), PS (8), QG (4), QH (2), QL (8), QS (4), QY (1), TG (1), TK (4), TL (1), TR (4), TS (4), VF (7), VG (1), VH (1), VK (1), VL (4), VS (4), VY (1)	IR (3), IY (2), VF (7), VY (1), PR (4), IVR (1), PL (5), IVY (1), VK (1), IF (2), VG (1), AG (9), MG (1), QG (4), TG (1), EG (4), NG (8), PG (2), VR (4), QK (6), NY (1), NF (1), NK (1), AR (2), EY (1), EK (1), PH (1), AQL (2), VTR (1), DY (1), IL (4)
Stem bromelain	**149:**	**84:**
	MA (2), KA (2), PA (2), HA (1), IA (2), WV (1), HL (2), PL (6), WR (1), YT (2), EG (4), ES (6), ET (2), EV (5), HR (3), HS (2), HT (1), HV (3), IL (5), IR (9), KF (2), KG (1), KR (2), KS (2), KT (4), MR (5), MV (1), NA (5), NF (1), NG (8), NL (3), NR (1), NV (2), PF (2), PG (3), PS (9), PT (1), QA (2), QG (6), QL (8), QS (5), QT (1), QV (2), YF (1), YL (3), YR (2), YS (2), YV (4)	IR (9), PR (4), YL (3), PL (6), IA (2), KR (2), IF (1), IG (2), HL (2), KG (1), DA (3), HG (1), QG (6), EG (4), EA (6), NG (8), PG (3), NKL (1), NF (1), KF (2), KA (2), EV (5), PT (1), YV (4), IL (5)

a The numbers in bold indicate the total number of sequences with given activity released from quinoa proteins by in silico enzymolysis.

b The numbers in the parentheses indicate the repetitions of the bioactive sequences.

### 3.3 Virtual screening of novel bioactive peptides

Herein, tripeptides released from in silico enzymolysis of quinoa proteins were further analyzed for the discovery of novel bioactive peptides with specific effect. Analysis of PeptideRanker predicted the theoretical bioactivity of peptides with the score values from 0.0222 to 0.9816 (Appendix S4). The top five peptides with high score were WCY, MAF, NMF, HPF, and MCG. Among them, WCY has been found in oat protein as an ACE inhibitory peptide (Bleakley, Hayes, O’ Shea, Gallagher, & Lafarga, 2017). However, the other four peptides, with no previously described bioactivity based on BIOPEP database and literatures, were subjected to in silico prediction of toxicity, solubility, and stability against the gastrointestinal digestion.

As shown in Table 5, the prediction has been given that all the selected peptides are nontoxic, and expected to be poorly soluble in water due to their high hydrophobic residues. To exert physiology effect, it is necessary that peptides survive gastrointestinal digestion. However, these four peptides exerted undesired low stability in simulative gastrointestinal digestion. As Udenigwe and Fogliano (2017) reported, encapsulation techniques need to be developed in the preparation of bioactive peptides in order to protect peptides from undesired degradation. It is also notable that two peptides were partly hydrolyzed, accompanying with the new generation of dipeptides PF and CG. PF is a DPP‐IV inhibitor documented in BIOPEP database, and CG has high theoretical bioactivity (0.9319) predicted by PeptideRanker. It indicated that these peptides could act as not only bioactive substance but also promising precursor.

**Table 5:** Predicted results of PeptideRanker score, toxicity, solubility, and stability against the gastrointestinal digestion of selected peptides

Peptide	Protein	Location	PeptideRanker score	Toxicity	Solubility	Simulated Digestion
MAF	13S‐1	f (1–3)	0.9676	Non‐Toxin	Poor	M‐A‐F
NMF	11S‐2	f (342–344)	0.9624	Non‐Toxin	Poor	N‐M‐F
HPF	11S‐2	f (97–99)	0.9502	Non‐Toxin	Poor	H‐PF
MCG	2S	f (128–130)	0.9502	Non‐Toxin	Poor	M‐CG

### 3.4 In vitro assessment of biological activity

In order to verify the bioactive effect of selected peptides, four chemically synthesized peptides were subjected to in vitro assessment of DPP‐IV and ACE inhibition activity. The assay results showed that all the peptides exhibited the positive ability in inhibiting DPP‐IV and ACE activity (Table 6). HPF exerted strongest DPP‐IV inhibition activity with IC_50_ value of 13.69 μg/ml, followed by MCG (45.95 μg/ml). MCG was the most potent ACE inhibitor with IC_50_ value of 6.48 μg/ml, followed by HPF (40.08 μg/ml). The inhibitory activity on DPP‐IV and ACE of MAF and NMF was comparatively lower despite the higher PeptideRanker score, which indicated that they may play a role in other biological functions.

**Table 6:** IC_50_ values (μg/ml) of chemically synthesized peptides in DPP‐IV and ACE inhibitory activities

Peptide	DPP‐IV inhibition	ACE inhibition
MAF	124.35 ± 1.75^a^	55.93 ± 1.09^b^
NMF	52.26 ± 0.83^b^	62.34 ± 1.21^a^
HPF	13.69 ± 0.76^d^	40.08 ± 0.59^c^
MCG	45.95 ± 0.91^c^	6.48 ± 0.12^d^

Note

Mean values followed by different letters in a column are significantly different (*p* < .01).

Nongonierma et al. (2015) reported that quinoa protein hydrolysate produced by papain has in vitro DPP‐IV inhibitory effect, while the peptide sequences have not been identified. Peptide IQAEGGLT, released from quinoa protein by pepsin‐pancreatin sequential digestion, has been reported to exert DPP‐IV inhibitory activity with an IC_50_ value of 267.81 μM (Vilcacundo et al., 2017). Compared with the bioactive peptides released by in silico proteolysis in this study, it is confirmed that the outcomes of in silico proteolysis and experimental enzymolysis were not an exact match (Nongonierma & FitzGerald, 2017; Tu, Cheng, Lu, & Du, 2018). In silico approach provides an alternative strategy for the investigation of novel bioactive peptides, but also has its limitations. Previous results showed that the products of enzymatic hydrolysis changed with the degree of hydrolysis, which was affected by kinds of factors, such as protein structural features, enzymatic activity, temperature, pH, hydrolysis time, and enzyme–substrate ratios (Fu et al., 2016; Han et al., 2019; Tu et al., 2018). However, the enzymolysis by in silico tools was rather idealistic, the digestion happened in every specific cutting site of enzymes, and it was carried out completely. Besides, in silico analysis was based on the current knowledge in BIOPEP database. The database is constantly updated, and the analysis results might be changed with new data.

## 4 CONCLUSION

Based on the selected protein sequences, our study revealed that the quinoa proteins contain various biological active peptides, especially DPP‐IV and ACE inhibitors. In silico proteolysis showed that papain has relative strong potential as an enzyme releasing DPP‐IV and ACE inhibitory peptides, although it exerts lower DH than stem bromelain. Furthermore, four novel bioactive tripeptides were selected by virtual screening and their bioactivities were confirmed using chemical synthesis and in vitro assay. In spite of some limitations in this in silico analysis, there is enough evidence to conclude that the quinoa protein is a promising precursor for production of bioactive peptides, and in silico proteolysis may serve the productive practice of the preparation of bioactive peptides.

## CONFLICTS OF INTEREST

The authors declare that they have no conflicts of interest.

## ETHICAL APPROVAL

There was no human or animal testing in this study.
