# Supplementary material for: Novel dipeptidyl peptidase‐IV and angiotensin‐I‐converting enzyme inhibitory peptides released from quinoa protein by in silico proteolysis
Source: Food Sci Nutr. 2020 Jan 27;8(3):1415–22. doi: 10.1002/fsn3.1423 (PMC7063354; doi:10.1002/fsn3.1423)
Supplement: Supplementary file 1 [file FSN3-8-1415-s001.docx]

**Selected protein sequences:**

1.

>XP_021758596.1 2S albumin-like [Chenopodium quinoa]

MAITKFVILAAVMAALVVMTHATIITTEVEIEDEFEQGRGSSSQCRRQLRSQWPNHCEQYMMQGMRRYMG

RDEEDDNQGREQYLEKCCDELKMMRPQCQCEAMKMMVEDKGMMHQQRMMEKAMNIPRMCGTMQRKCRMSK

ME

2

>AAS67036.1 11S seed storage globulin [Chenopodium quinoa]

MAKSTTTLFLLSCSIALVLLNGCMGQGRMREMQGNECQIDRLTALEPTNRIQAEGGLTEVWDTQDQQFQC

SGVSVIRRTIEPNGLLLPSFTSGPELIYIEQGNGISGLMIPGCPETFESMSQESWREGMKRGMRGGRFQD

QHQKIRHLRQGHIFAMPAGVAHWAYNTGNEPLVAVILIDTSNHANQLDKDYPKRFYLAGKPQQEHSRHQH

RGGESQRGERGSGGNVFSGLGTKTIAQSFGVSEDIAEKLQAEQDERGNIVLVQEGLHVIKPPSSRSYDDE

REQRRHRSPRSNGLEETICSARLSENIDEPSKADVYSPEAGRLTTLNSFNLPILSNLRLSAEKGVLYRNA

IMAPHYNLNAHSIIYGVRGRGRIQIVNAQGNSVFDDELRQGQLVVVPQNFAVVKQAGEEGFEWIAFKTCE

NALFQTLAGRTSAIRAMPLEVISNIYQISREQAYRLKFSRSETTLFRPENQGRQRRDLAA

3

>XP_021770184.1 11S globulin seed storage protein 2-like [Chenopodium quinoa]

MGGTKILVALSLCLMVSSALGQGSQKRLSVRIQLLLIYQAQQCRINRLTSSEPNQRVECEGGLIELWDET

EEQFQCSGIHAMRVTVQHNSLSLPNFHPFPRLVYIERGEGILGVTFPGCPETYDSSGRQEEGIRGDEQRE

FGHQKDLHQKVHRFTRGDIIAIPPGAVHWCYNDGNEEVVTVIVNDLNNPSNQLDQTFRSFYLAGGVERSS

EQRGKHTQQQFNNILRPFDPELLSEAFDVPEDLVRKMQQTENRGLIVRVDKGEMRILSPGSEQDYDDERR

RKYVGLDVNGLEETICTMRLRHNLDNRREADVYSRHGGRLNIVNEHKLPILRHLDMSVEKGNMFPNTIYS

PHWAVNSHSVVYVTRGEAHVQVVGNNGQSVMDDRVNEGEMFVIPQYFTVSVKAGSNGFEYVSFKTTSSPM

KSPMVGYTSVLRAMPVQVLTNAYQISPSEAHQLKYNREHQTFFLPSRGGKSRRF

4

>XP_021752233.1 13S globulin seed storage protein 1-like [Chenopodium quinoa]

MAFTTTNNNALLFWVPLCLLVFLISPSLAQLPLLQRQPQQPRGQQWQHDCDIQQLQAAEPTHRLRAEAGV

IEVWESNSEQFRCAGVAAVRYVIEPKGLLLPSYTNAPYVTYVTQGRGIQGVIVPGCPETFESPRGSGSDT

TREGQRDQHQKVFRVQEGDVIGSPAGVVQWTYNDGDAPIVSVTLLDLSNPNNQLDLNFRSFYLAGDPQGG

QERRPKEVAGKNIFNGFDDEMLADAFNVDTETIRSMKAENDERGSIIRVERDLEILSPEWDDTEEERTRR

LNGLEQTLCSLIFKQNIDRPSLADVFTKHGGRINTLNGHKLPLLQYLQLSVERGVLYKNALMTPHWNINA

HSIIYITRGTGWIQVARENGRLVFDDRVQEGQLLVVPQNFVVVKKAEQEGLKWVSFKTNDNAMISPLAGK

LSAIRGMPEEVLMNSYDMSRDEVRRLKYGREELSLFSPRTRSF

5

>XP_021752668.1 13S globulin seed storage protein 2-like [Chenopodium quinoa]

MSRVFLLPLALTLLILSPTSLAQLGFQLGQSPFLPSGQSSPQHSRLQRGQQALNDCQINQLSANEPSIRI

QAEAGITEVWDPKEQQEFQCAGVTVIRREIEPKGLLLPHYNNAPSISYVIRGRGLLGLSSLGCADTYESG

SPEFFSEESRRSERFEESRRSERGSEEMRDQHQKVRRFHKGHVIGLPAGVSKWVYNDGEDRLTIVTLYDT

NNFQNQLDDNLRSFFLAGNPQGRGGDQSGRQHESSRRHTRGGQEEMGQNILSGFDKQLLADAFEVESDTI

SKIQGENDDRGAIIRVESGELEMLIPEWDQEEQRSERHHRGGGSERSEEEERSERHHRGGRGRQSESSRP

HNGIEQTLCSARLSVNIDNPERADVFNPQGGRLTNINSNKLPILNYLRLSAEKVNLYKNAIMTPNWKINA

HSIVYFTKGSGRVQIANHEGELVFDDMVQEGQLVVVPQNFVVLKRAGQDGLEWVALLTNDNAMSSPLAGR

ISAIRGMPIEVVMNSYKLSREEAQRLKYGRQELSVFSPSKRSERRGDEYAIV
