# Supplementary material for: Novel dipeptidyl peptidase‐IV and angiotensin‐I‐converting enzyme inhibitory peptides released from quinoa protein by in silico proteolysis
Source: Food Sci Nutr. 2020 Jan 27;8(3):1415–22. doi: 10.1002/fsn3.1423 (PMC7063354; doi:10.1002/fsn3.1423)
Supplement: Supplementary file 2 [file FSN3-8-1415-s002.docx]

***In silico* proteolysis of quinoa proteins by papain, ficin and stem bromelain**

**2S albumin-like**

**1.1 Enzymolysis by Papain:**

M - AIT - KF - VIL - A - AVM - A - AL - VVMT - H - AT - IIT - T - EVEIEDEF - E - QG - R - G - SSS - QCR - R - QL - R - S - QWPNHCE - QYMM - QG - MR - R - YMG - R - DEEDDN - QG - R - E - QYL - EKCCDEL - KMMR - P - QC - QCE - AMKMMVEDKG - MMH - Q - QR - MMEK - AMNIPR - MCG - T - M - QR - KCR - MSKME

**1.2 Enzymolysis by Ficin:**

MAITK - F - VIL - AAVMAAL - VVMTH - ATIITTEVEIEDEF - EQG - R - G - S - S - S - QCR - R - QL - R - S - QWPNH - CEQY - MMQG - MR - R - Y - MG - R - DEEDDNQG - R - EQY - L - EK - CCDEL - K - MMR - PQCQCEAMK - MMVEDK - G - MMH - QQR - MMEK - AMNIPR - MCG - TMQR - K - CR - MS - K - ME

**1.3 Enzymolysis by Stem bromelain:**

MA - IT - KF - V - IL - A - A - V - MA - A - L - V - V - MT - HA - T - IIT - T - EV - EIEDEF - EQG - R - G - S - S - S - QCR - R - QL - R - S - QWPNHCEQYMMQG - MR - R - YMG - R - DEEDDNQG - R - EQYL - EKCCDEL - KMMR - PQCQCEA - MKMMV - EDKG - MMHQQR - MMEKA - MNIPR - MCG - T - MQR - KCR - MS - KME

**11S seed storage globulin**

**2.1 Enzymolysis by Papain:**

M - AKST - T - T - L - F - L - L - SCSI - AL - VL - L - NG - CMG - QG - R - MR - EM - QG - NEC - QIDR - L - T - AL - EPT - NR - I - Q - AEG - G - L - T - EVWDT - QD - Q - QF - QC SG - VSVIR - R - T - IEPNG - L - L - L - PSF - T - SG - PEL - IYIE - QG - NG - ISG - L - MIPG - CPET - F - ESMS - QESWR - EG - MKR - G - MR - G - G - R - F - QD - QH - QKIR - HL - R - QG - HIF - AMP - AG - V - AHW - AYNT - G - NEPL - V - AVIL - IDT - SNH - AN - QL - DKDYPKR - F - YL - AG - KP - Q - QEHSR - H - QH R - G - G - ES - QR - G - ER - G - SG - G - NVF - SG - L - G - T - KT - I - A - QSF - G - VSEDI - AEKL - Q - AE - QDER - G - NIVL - V - QEG - L - HVIKPPSSR - SYDDE R - E - QR - R - HR - SPR - SNG - L - EET - ICS - AR - L - SENIDEPSK - ADVYSPE - AG - R - L - T - T - L - NSF - NL - PIL - SNL - R - L - S - AEKG - VL - YR - N - A IM - APHYNL - N - AHSIIYG - VR - G - R - G - R - I - QIVN - A - QG - NSVF - DDEL - R - QG - QL - VVVP - QNF - AVVK - Q - AG - EEG - F - EWI - AF - KT - CE N - AL - F - QT - L - AG - R - T - S - AIR - AMPL - EVISNIY - QISR - E - Q - AYR - L - KF - SR - SET - T - L - F - R - PEN - QG - R - QR - R - DL - A - A

**2.2 Enzymolysis by Ficin**

MAK - S - TTTL - F - L - L - S - CS - IAL - VL - L - NG - CMG - QG - R - MR - EMQG - NECQIDR - L - TAL - EPTNR - IQAEG - G - L - TEVWDTQDQQF - QC S - G - VS - VIR - R - TIEPNG - L - L - L - PS - F - TS - G - PEL - IY - IEQG - NG - IS - G - L - MIPG - CPETF - ES - MS - QES - WR - EG - MK - R - G - MR - G - G - R - F - QD QH - QK - IR - H - L - R - QG - H - IF - AMPAG - VAH - WAY - NTG - NEPL - VAVIL - IDTS - NH - ANQL - DK - DY - PK - R - F - Y - L - AG - K - PQQEH - S - R - H - QH - R - G - G - ES - QR - G - ER - G - S - G - G - NVF - S - G - L - G - TK - TIAQS - F - G - VS - EDIAEK - L - QAEQDER - G - NIVL - VQEG - L - H - VIK - PPS - S - R - S - Y - DDE R - EQR - R - H - R - S - PR - S - NG - L - EETICS - AR - L - S - ENIDEPS - K - ADVY - S - PEAG - R - L - TTL - NS - F - NL - PIL - S - NL - R - L - S - AEK - G - VL - Y - R - NA IMAPH - Y - NL - NAH - S - IIY - G - VR - G - R - G - R - IQIVNAQG - NS - VF - DDEL - R - QG - QL - VVVPQNF - AVVK - QAG - EEG - F - EWIAF - K - TCE NAL - F - QTL - AG - R - TS - AIR - AMPL - EVIS - NIY - QIS - R - EQAY - R - L - K - F - S - R - S - ETTL - F - R - PENQG - R - QR - R - DL - AA

**2.3 Enzymolysis by Stem bromelain:**

MA - KS - T - T - T - L - F - L - L - S - CS - IA - L - V - L - L - NG - CMG - QG - R - MR - EMQG - NECQIDR - L - T - A - L - EPT - NR - IQA - EG - G - L - T - EV - WDT - QDQQF - QC S - G - V - S - V - IR - R - T - IEPNG - L - L - L - PS - F - T - S - G - PEL - IYIEQG - NG - IS - G - L - MIPG - CPET - F - ES - MS - QES - WR - EG - MKR - G - MR - G - G - R - F - QD QHQKIR - HL - R - QG - HIF - A - MPA - G - V - A - HWA - YNT - G - NEPL - V - A - V - IL - IDT - S - NHA - NQL - DKDYPKR - F - YL - A - G - KPQQEHS - R - HQH R - G - G - ES - QR - G - ER - G - S - G - G - NV - F - S - G - L - G - T - KT - IA - QS - F - G - V - S - EDIA - EKL - QA - EQDER - G - NIV - L - V - QEG - L - HV - IKPPS - S - R - S - YDDE R - EQR - R - HR - S - PR - S - NG - L - EET - ICS - A - R - L - S - ENIDEPS - KA - DV - YS - PEA - G - R - L - T - T - L - NS - F - NL - PIL - S - NL - R - L - S - A - EKG - V - L - YR - NA - IMA - PHYNL - NA - HS - IIYG - V - R - G - R - G - R - IQIV - NA - QG - NS - V - F - DDEL - R - QG - QL - V - V - V - PQNF - A - V - V - KQA - G - EEG - F - EWIA - F - KT - CE NA - L - F - QT - L - A - G - R - T - S - A - IR - A - MPL - EV - IS - NIYQIS - R - EQA - YR - L - KF - S - R - S - ET - T - L - F - R - PENQG - R - QR - R - DL - A - A -

**11S globulin seed storage protein 2-like**

**3.1 Enzymolysis by Papain:**

MG - G - T - KIL - V - AL - SL - CL - MVSS - AL - G - QG - S - QKR - L - SVR - I - QL - L - L - IY - Q - A - Q - QCR - INR - L - T - SSEPN - QR - VECEG - G - L - IEL - WDET - EE - QF - QCSG - IH - AMR - VT - V - QHNSL - SL - PNF - HPF - PR - L - VYIER - G - EG - IL - G - VT - F - PG - CPET - YDSSG - R - QEEG - IR - G - DE - QR - E F - G - H - QKDL - H - QKVHR - F - T - R - G - DII - AIPPG - AVHWCYNDG - NEEVVT - VIVNDL - NNPSN - QL - D - QT - F - R - SF - YL - AG - G - VER - SS E - QR - G - KHT - Q - Q - QF - NNIL - R - PF - DPEL - L - SE - AF - DVPEDL - VR - KM - Q - QT - ENR - G - L - IVR - VDKG - EMR - IL - SPG - SE - QDYDDER - R - R - KYVG - L - DVNG - L - EET - ICT - MR - L - R - HNL - DNR - R - E - ADVYSR - HG - G - R - L - NIVNEHKL - PIL - R - HL - DMSVEKG - NMF - PNT - IYS PHW - AVNSHSVVYVT - R - G - E - AHV - QVVG - NNG - QSVMDDR - VNEG - EMF - VIP - QYF - T - VSVK - AG - SNG - F - EYVSF - KT - T - SSPM KSPMVG - YT - SVL - R - AMPV - QVL - T - N - AY - QISPSE - AH - QL - KYNR - EH - QT - F - F - L - PSR - G - G - KSR - R - F

**3.2 Enzymolysis by Ficin:**

MG - G - TK - IL - VAL - S - L - CL - MVS - S - AL - G - QG - S - QK - R - L - S - VR - IQL - L - L - IY - QAQQCR - INR - L - TS - S - EPNQR - VECEG - G - L - IEL - WDET EEQF - QCS - G - IH - AMR - VTVQH - NS - L - S - L - PNF - H - PF - PR - L - VY - IER - G - EG - IL - G - VTF - PG - CPETY - DS - S - G - R - QEEG - IR - G - DEQR - E F - G - H - QK - DL - H - QK - VH - R - F - TR - G - DIIAIPPG - AVH - WCY - NDG - NEEVVTVIVNDL - NNPS - NQL - DQTF - R - S - F - Y - L - AG - G - VER - S - S - EQR - G - K - H - TQQQF - NNIL - R - PF - DPEL - L - S - EAF - DVPEDL - VR - K - MQQTENR - G - L - IVR - VDK - G - EMR - IL - S - PG - S - EQDY - DDER - R - R - K - Y - VG - L - DVNG - L - EETICTMR - L - R - H - NL - DNR - R - EADVY - S - R - H - G - G - R - L - NIVNEH - K - L - PIL - R - H - L - DMS - VEK - G - NMF - PNTIY - S - PH - WAVNS - H - S - VVY - VTR - G - EAH - VQVVG - NNG - QS - VMDDR - VNEG - EMF - VIPQY - F - TVS - VK - AG - S - NG - F - EY - VS - F - K - TTS - S - PM K - S - PMVG - Y - TS - VL - R - AMPVQVL - TNAY - QIS - PS - EAH - QL - K - Y - NR - EH - QTF - F - L - PS - R - G - G - K - S - R - R - F -

**3.3 Enzymolysis by Stem bromelain:**

MG - G - T - KIL - V - A - L - S - L - CL - MV - S - S - A - L - G - QG - S - QKR - L - S - V - R - IQL - L - L - IYQA - QQCR - INR - L - T - S - S - EPNQR - V - ECEG - G - L - IEL - WDET - EEQF - QCS - G - IHA - MR - V - T - V - QHNS - L - S - L - PNF - HPF - PR - L - V - YIER - G - EG - IL - G - V - T - F - PG - CPET - YDS - S - G - R - QEEG - IR - G - DEQR - E F - G - HQKDL - HQKV - HR - F - T - R - G - DIIA - IPPG - A - V - HWCYNDG - NEEV - V - T - V - IV - NDL - NNPS - NQL - DQT - F - R - S - F - YL - A - G - G - V - ER - S - S - EQR - G - KHT - QQQF - NNIL - R - PF - DPEL - L - S - EA - F - DV - PEDL - V - R - KMQQT - ENR - G - L - IV - R - V - DKG - EMR - IL - S - PG - S - EQDYDDER - R - R - KYV - G - L - DV - NG - L - EET - ICT - MR - L - R - HNL - DNR - R - EA - DV - YS - R - HG - G - R - L - NIV - NEHKL - PIL - R - HL - DMS - V - EKG - NMF - PNT - IYS - PHWA - V - NS - HS - V - V - YV - T - R - G - EA - HV - QV - V - G - NNG - QS - V - MDDR - V - NEG - EMF - V - IPQYF - T - V - S - V - KA - G - S - NG - F - EYV - S - F - KT - T - S - S - PM KS - PMV - G - YT - S - V - L - R - A - MPV - QV - L - T - NA - YQIS - PS - EA - HQL - KYNR - EHQT - F - F - L - PS - R - G - G - KS - R - R - F -

**13S globulin seed storage protein 1-like**

**4.1 Enzymolysis by Papain:**

M - AF - T - T - T - NNN - AL - L - F - WVPL - CL - L - VF - L - ISPSL - A - QL - PL - L - QR - QP - Q - QPR - G - Q - QW - QHDCDI - Q - QL - Q - A - AEPT - HR - L - R - AE - AG - V IEVWESNSE - QF - R - C - AG - V - A - AVR - YVIEPKG - L - L - L - PSYT - N - APYVT - YVT - QG - R - G - I - QG - VIVPG - CPET - F - ESPR - G - SG - SDT - T - R - EG - QR - D - QH - QKVF - R - V - QEG - DVIG - SP - AG - VV - QWT - YNDG - D - APIVSVT - L - L - DL - SNPNN - QL - DL - NF - R - SF - YL - AG - DP - QG - G - - QER - R - PKEV - AG - KNIF - NG - F - DDEML - AD - AF - NVDT - ET - IR - SMK - AENDER - G - SIIR - VER - DL - EIL - SPEWDDT - EEER - T - R - R - L - NG - L - E - QT - L - CSL - IF - K - QNIDR - PSL - ADVF - T - KHG - G - R - INT - L - NG - HKL - PL - L - QYL - QL - SVER - G - VL - YKN - AL - MT - PHWNIN - A HSIIYIT - R - G - T - G - WI - QV - AR - ENG - R - L - VF - DDR - V - QEG - QL - L - VVP - QNF - VVVKK - AE - QEG - L - KWVSF - KT - NDN - AMISPL - AG - K L - S - AIR - G - MPEEVL - MNSYDMSR - DEVR - R - L - KYG - R - EEL - SL - F - SPR - T - R - SF -

**4.2 Enzymolysis by Ficin:**

MAF - TTTNNNAL - L - F - WVPL - CL - L - VF - L - IS - PS - L - AQL - PL - L - QR - QPQQPR - G - QQWQH - DCDIQQL - QAAEPTH - R - L - R - AEAG - V IEVWES - NS - EQF - R - CAG - VAAVR - Y - VIEPK - G - L - L - L - PS - Y - TNAPY - VTY - VTQG - R - G - IQG - VIVPG - CPETF - ES - PR - G - S - G - S - DT TR - EG - QR - DQH - QK - VF - R - VQEG - DVIG - S - PAG - VVQWTY - NDG - DAPIVS - VTL - L - DL - S - NPNNQL - DL - NF - R - S - F - Y - L - AG - DPQG - G - QER - R - PK - EVAG - K - NIF - NG - F - DDEML - ADAF - NVDTETIR - S - MK - AENDER - G - S - IIR - VER - DL - EIL - S - PEWDDTEEER - TR - R - L - NG - L - EQTL - CS - L - IF - K - QNIDR - PS - L - ADVF - TK - H - G - G - R - INTL - NG - H - K - L - PL - L - QY - L - QL - S - VER - G - VL - Y - K - NAL - MTPH - WNINA H - S - IIY - ITR - G - TG - WIQVAR - ENG - R - L - VF - DDR - VQEG - QL - L - VVPQNF - VVVK - K - AEQEG - L - K - WVS - F - K - TNDNAMIS - PL - AG - K - L - S - AIR - G - MPEEVL - MNS - Y - DMS - R - DEVR - R - L - K - Y - G - R - EEL - S - L - F - S - PR - TR - S - F -

**4.3 Enzymolysis by Stem bromelain:**

MA - F - T - T - T - NNNA - L - L - F - WV - PL - CL - L - V - F - L - IS - PS - L - A - QL - PL - L - QR - QPQQPR - G - QQWQHDCDIQQL - QA - A - EPT - HR - L - R - A - EA - G - V - IEV - WES - NS - EQF - R - CA - G - V - A - A - V - R - YV - IEPKG - L - L - L - PS - YT - NA - PYV - T - YV - T - QG - R - G - IQG - V - IV - PG - CPET - F - ES - PR - G - S - G - S - DT - T - R - EG - QR - DQHQKV - F - R - V - QEG - DV - IG - S - PA - G - V - V - QWT - YNDG - DA - PIV - S - V - T - L - L - DL - S - NPNNQL - DL - NF - R - S - F - YL - A - G - DPQG - G - QER - R - PKEV - A - G - KNIF - NG - F - DDEML - A - DA - F - NV - DT - ET - IR - S - MKA - ENDER - G - S - IIR - V - ER - DL - EIL - S - PEWDDT - EEER - T - R - R - L - NG - L - EQT - L - CS - L - IF - KQNIDR - PS - L - A - DV - F - T - KHG - G - R - INT - L - NG - HKL - PL - L - QYL - QL - S - V - ER - G - V - L - YKNA - L - MT - PHWNINA - HS - IIYIT - R - G - T - G - WIQV - A - R - ENG - R - L - V - F - DDR - V - QEG - QL - L - V - V - PQNF - V - V - V - KKA - EQEG - L - KWV - S - F - KT - NDNA - MIS - PL - A - G - K L - S - A - IR - G - MPEEV - L - MNS - YDMS - R - DEV - R - R - L - KYG - R - EEL - S - L - F - S - PR - T - R - S - F -

**13S globulin seed storage protein 2-like**

**5.1 Enzymolysis by Papain:**

MSR - VF - L - L - PL - AL - T - L - L - IL - SPT - SL - A - QL - G - F - QL - G - QSPF - L - PSG - QSSP - QHSR - L - QR - G - Q - Q - AL - NDC - QIN - QL - S - ANEPSIR - I - Q - AE - AG - IT - EVWDPKE - Q - QEF - QC - AG - VT - VIR - R - EIEPKG - L - L - L - PHYNN - APSISYVIR - G - R - G - L - L - G - L - SSL - G - C - ADT - YESG - SPEF - F - SEESR - R - SER - F - EESR - R - SER - G - SEEMR - D - QH - QKVR - R - F - HKG - HVIG - L - P - AG - VSKWVYNDG - EDR - L - T - IVT - L - YDT - NNF - QN - QL - DDNL - R - SF - F - L - AG - NP - QG - R - G - G - D - QSG - R - QHESSR - R - HT - R - G - G - QEEMG - QNIL - SG - F - DK - QL - L - AD - AF - EVESDT - I SKI - QG - ENDDR - G - AIIR - VESG - EL - EML - IPEWD - QEE - QR - SER - HHR - G - G - G - SER - SEEEER - SER - HHR - G - G - R - G - R - QSESSR - P HNG - IE - QT - L - CS - AR - L - SVNIDNPER - ADVF - NP - QG - G - R - L - T - NINSNKL - PIL - NYL - R - L - S - AEKVNL - YKN - AIMT - PNWKIN - A HSIVYF - T - KG - SG - R - V - QI - ANHEG - EL - VF - DDMV - QEG - QL - VVVP - QNF - VVL - KR - AG - QDG - L - EWV - AL - L - T - NDN - AMSSPL - AG - R - IS - AIR - G - MPIEVVMNSYKL - SR - EE - A - QR - L - KYG - R - QEL - SVF - SPSKR - SER - R - G - DEY - AIV

**5.2 Enzymolysis by Ficin:**

MS - R - VF - L - L - PL - AL - TL - L - IL - S - PTS - L - AQL - G - F - QL - G - QS - PF - L - PS - G - QS - S - PQH - S - R - L - QR - G - QQAL - NDCQINQL - S - ANEPS - IR - I QAEAG - ITEVWDPK - EQQEF - QCAG - VTVIR - R - EIEPK - G - L - L - L - PH - Y - NNAPS - IS - Y - VIR - G - R - G - L - L - G - L - S - S - L - G - CADTY - ES - G - S - PEF - F - S - EES - R - R - S - ER - F - EES - R - R - S - ER - G - S - EEMR - DQH - QK - VR - R - F - H - K - G - H - VIG - L - PAG - VS - K - WVY - NDG - EDR - L - TIVTL - Y - DT NNF - QNQL - DDNL - R - S - F - F - L - AG - NPQG - R - G - G - DQS - G - R - QH - ES - S - R - R - H - TR - G - G - QEEMG - QNIL - S - G - F - DK - QL - L - ADAF - EVES - DTI S - K - IQG - ENDDR - G - AIIR - VES - G - EL - EML - IPEWDQEEQR - S - ER - H - H - R - G - G - G - S - ER - S - EEEER - S - ER - H - H - R - G - G - R - G - R - QS - ES - S - R - P H - NG - IEQTL - CS - AR - L - S - VNIDNPER - ADVF - NPQG - G - R - L - TNINS - NK - L - PIL - NY - L - R - L - S - AEK - VNL - Y - K - NAIMTPNWK - INA H - S - IVY - F - TK - G - S - G - R - VQIANH - EG - EL - VF - DDMVQEG - QL - VVVPQNF - VVL - K - R - AG - QDG - L - EWVAL - L - TNDNAMS - S - PL - AG - R - IS - AIR - G - MPIEVVMNS - Y - K - L - S - R - EEAQR - L - K - Y - G - R - QEL - S - VF - S - PS - K - R - S - ER - R - G - DEY - AIV

**5.3 Enzymolysis by Stem bromelain:**

MS - R - V - F - L - L - PL - A - L - T - L - L - IL - S - PT - S - L - A - QL - G - F - QL - G - QS - PF - L - PS - G - QS - S - PQHS - R - L - QR - G - QQA - L - NDCQINQL - S - A - NEPS - IR - I QA - EA - G - IT - EV - WDPKEQQEF - QCA - G - V - T - V - IR - R - EIEPKG - L - L - L - PHYNNA - PS - IS - YV - IR - G - R - G - L - L - G - L - S - S - L - G - CA - DT - YES - G - S - PEF - F - S - EES - R - R - S - ER - F - EES - R - R - S - ER - G - S - EEMR - DQHQKV - R - R - F - HKG - HV - IG - L - PA - G - V - S - KWV - YNDG - EDR - L - T - IV - T - L - YDT - NNF - QNQL - DDNL - R - S - F - F - L - A - G - NPQG - R - G - G - DQS - G - R - QHES - S - R - R - HT - R - G - G - QEEMG - QNIL - S - G - F - DKQL - L - A - DA - F - EV - ES - DT - I S - KIQG - ENDDR - G - A - IIR - V - ES - G - EL - EML - IPEWDQEEQR - S - ER - HHR - G - G - G - S - ER - S - EEEER - S - ER - HHR - G - G - R - G - R - QS - ES - S - R - P HNG - IEQT - L - CS - A - R - L - S - V - NIDNPER - A - DV - F - NPQG - G - R - L - T - NINS - NKL - PIL - NYL - R - L - S - A - EKV - NL - YKNA - IMT - PNWKINA - HS - IV - YF - T - KG - S - G - R - V - QIA - NHEG - EL - V - F - DDMV - QEG - QL - V - V - V - PQNF - V - V - L - KR - A - G - QDG - L - EWV - A - L - L - T - NDNA - MS - S - PL - A - G - R - IS - A - IR - G - MPIEV - V - MNS - YKL - S - R - EEA - QR - L - KYG - R - QEL - S - V - F - S - PS - KR - S - ER - R - G - DEYA - IV -
