# Supplementary material for: Novel dipeptidyl peptidase‐IV and angiotensin‐I‐converting enzyme inhibitory peptides released from quinoa protein by in silico proteolysis
Source: Food Sci Nutr. 2020 Jan 27;8(3):1415–22. doi: 10.1002/fsn3.1423 (PMC7063354; doi:10.1002/fsn3.1423)
Supplement: Supplementary file 3 [file FSN3-8-1415-s003.docx]

**File S3. Cleavage sites of enzymes used for *in silico*** **enzymolysis. (from BIOPEP)**

| Enzyme | EC number | Cutting sites | |
| --- | --- | --- | --- |
|  |  | C-terminus | N-terminus |
| Papain | EC 3.4.22.2 | R, F, L, G, T | Q, A |
| Ficin | EC 3.4.22.3 | K, F, Y, G, S, L, R, H | - |
| Stem bromelain | EC 3.4.22.32 | V, A, T, L, R, G, S, F | - |
