# Supplementary material for: Novel dipeptidyl peptidase‐IV and angiotensin‐I‐converting enzyme inhibitory peptides released from quinoa protein by in silico proteolysis
Source: Food Sci Nutr. 2020 Jan 27;8(3):1415–22. doi: 10.1002/fsn3.1423 (PMC7063354; doi:10.1002/fsn3.1423)
Supplement: Supplementary file 4 [file FSN3-8-1415-s004.docx]

**PeptideRanker scores of tripeptides released from *in silico* enzymolysis of quinoa proteins**

| **Score** | **Peptide** | **Score** | **Peptide** | **Score** | **Peptide** | **Score** | **Peptide** | **Score** | **Peptide** |
| --- | --- | --- | --- | --- | --- | --- | --- | --- | --- |
| 0.981644 | WCY | 0.539679 | PSL | 0.244517 | SNL | 0.131234 | SVL | 0.077798 | DQS |
| 0.967577 | MAF | 0.511759 | KCR | 0.239945 | DDR | 0.131094 | TAL | 0.075907 | EDR |
| 0.962371 | NMF | 0.500704 | PSR | 0.239210 | SSL | 0.129634 | QTL | 0.074540 | KYV |
| 0.950199 | HPF | 0.497995 | NVF | 0.237518 | HNL | 0.129560 | DQH | 0.073992 | KQA |
| 0.950178 | MCG | 0.464805 | IMA | 0.235253 | PEL | 0.129545 | AVR | 0.072804 | DQT |
| 0.947827 | CMG | 0.464540 | MKR | 0.234644 | NAL | 0.127235 | VAL | 0.072584 | NIV |
| 0.937503 | PNF | 0.446607 | QCA | 0.232905 | IQL | 0.126256 | NTG | 0.071682 | KKA |
| 0.922363 | MPL | 0.433890 | AYR | 0.232795 | AQL | 0.125921 | YDT | 0.070357 | EQY |
| 0.920402 | PSF | 0.422909 | EAF | 0.232451 | NNG | 0.123492 | PEN | 0.069522 | EKG |
| 0.910067 | MMR | 0.421681 | QYL | 0.231650 | NIY | 0.123023 | ITR | 0.068929 | EAH |
| 0.891836 | QYF | 0.421425 | KWV | 0.229395 | PIV | 0.122924 | SVR | 0.068740 | EQR |
| 0.890637 | WAY | 0.413623 | EWI | 0.227895 | VIP | 0.122286 | IQA | 0.068100 | ENR |
| 0.855408 | YMG | 0.400761 | QEF | 0.227203 | EWV | 0.121262 | IIT | 0.065324 | YVT |
| 0.853636 | MMH | 0.397029 | WVS | 0.224682 | NDG | 0.120783 | QVL | 0.064990 | TTL |
| 0.847715 | AMP | 0.394263 | NDC | 0.221017 | HKG | 0.120387 | YNT | 0.062562 | YES |
| 0.847307 | AHW | 0.374970 | NYL | 0.220448 | HQL | 0.120325 | IVY | 0.062181 | VTL |
| 0.809917 | MPA | 0.372793 | ICS | 0.219615 | KHG | 0.119348 | KME | 0.061912 | KHT |
| 0.806634 | EMF | 0.353100 | ICT | 0.213706 | INR | 0.116790 | VIR | 0.061594 | SSE |
| 0.781654 | NIF | 0.350513 | SMK | 0.213104 | QSG | 0.116007 | IVR | 0.061157 | VVL |
| 0.778100 | HIF | 0.348223 | EQF | 0.210658 | DNR | 0.111486 | AEG | 0.059637 | EKL |
| 0.769301 | QNF | 0.345645 | PQH | 0.207321 | NDL | 0.109055 | AIT | 0.056238 | VTY |
| 0.756909 | HWA | 0.345402 | MIS | 0.206752 | PNT | 0.106219 | QIS | 0.056091 | VIK |
| 0.730422 | CAG | 0.341871 | IMT | 0.201093 | QQR | 0.106135 | VNL | 0.054911 | VTR |
| 0.723457 | PEF | 0.320815 | KYG | 0.192577 | DKG | 0.104183 | EIL | 0.054347 | VVY |
| 0.717769 | NNF | 0.319283 | QCS | 0.192453 | KIL | 0.104132 | AIV | 0.047697 | IVT |
| 0.712391 | WVY | 0.319122 | DMS | 0.190323 | HKL | 0.101701 | QQA | 0.046850 | EYV |
| 0.700230 | QSF | 0.317865 | VTF | 0.183399 | QCE | 0.101618 | SSS | 0.045779 | EQA |
| 0.697499 | SPG | 0.314094 | PYV | 0.181781 | NQL | 0.100303 | IEL | 0.044590 | EEG |
| 0.688815 | AMR | 0.312400 | EML | 0.180278 | DII | 0.096014 | VVP | 0.043970 | VDK |
| 0.686594 | NSF | 0.307571 | MAK | 0.177213 | IYS | 0.095599 | EPT | 0.042718 | AEK |
| 0.680792 | PAG | 0.307096 | IIY | 0.175815 | IHA | 0.095373 | AHV | 0.042518 | VER |
| 0.667069 | PMK | 0.306272 | IAL | 0.164932 | VIG | 0.093784 | AVH | 0.041968 | SET |
| 0.643288 | QCR | 0.299341 | AVM | 0.159349 | NEC | 0.093433 | QEG | 0.041258 | QES |
| 0.641797 | PIL | 0.295430 | QAG | 0.157601 | QKR | 0.093130 | NNN | 0.039385 | EEL |
| 0.623557 | QWT | 0.292982 | MKA | 0.151993 | SNH | 0.091891 | ADT | 0.035704 | IEV |
| 0.617487 | SPR | 0.289821 | WES | 0.151058 | CEN | 0.091547 | NDN | 0.033327 | TTS |
| 0.603646 | PPS | 0.289458 | AIR | 0.150864 | MVS | 0.088921 | SDT | 0.032287 | TVS |
| 0.601262 | CSL | 0.286486 | IIR | 0.149225 | NKL | 0.088385 | IDT | 0.032199 | DEV |
| 0.593031 | WDT | 0.286009 | EMR | 0.143878 | NAH | 0.087922 | SER | 0.032179 | EQT |
| 0.586522 | PMV | 0.285710 | YKL | 0.142915 | PTS | 0.086925 | ENG | 0.029857 | EEA |
| 0.585120 | QPR | 0.285281 | ISG | 0.142306 | QIA | 0.086705 | IER | 0.029687 | QEE |
| 0.575639 | MPV | 0.281192 | SNG | 0.137944 | YKN | 0.085956 | VAH | 0.028244 | VES |
| 0.573515 | PSG | 0.272281 | IQG | 0.137741 | NHA | 0.085854 | NEG | 0.027835 | EES |
| 0.573302 | SVF | 0.271571 | MNS | 0.137620 | VIL | 0.083948 | QEL | 0.024258 | EKV |
| 0.571786 | AIM | 0.269398 | SPT | 0.136657 | YDS | 0.083465 | INT | 0.023735 | VEK |
| 0.565724 | QTF | 0.258747 | HHR | 0.135995 | PEA | 0.083198 | EQG | 0.0221731 | EET |
| 0.554004 | MSR | 0.256327 | QHR | 0.133226 | KSR | 0.081694 | QER |  |  |
| 0.540802 | MQR | 0.246360 | QDG | 0.131826 | QIN | 0.080027 | DEY |  |  |
